# Diversity, Endemism, and Conservation Status of the Herpetofauna of the Sierra Madre Occidental in Mexico with Comparison to Neighboring Biogeographic Provinces

**DOI:** 10.3390/ani15091278

**Published:** 2025-04-30

**Authors:** Geoffrey R. Smith, Julio A. Lemos-Espinal

**Affiliations:** 1 Department of Biology, Denison University, Granville, OH 43023, USA; smithg@denison.edu; 2 Laboratorio de Ecología-UBIPRO, Facultad de Estudios Superiores Iztacala Universidad Nacional Autónoma de México, Avenida los Barrios 1, Los Reyes Iztacala, Tlalnepantla 54090, Mexico

**Keywords:** amphibian endemism, biogeography, conservation threats, habitat fragmentation, IUCN Red List, reptile species richness

## Abstract

The Sierra Madre Occidental (SMO) is a biogeographic province of Mexico that serves as a corridor for the northward expansion of subtropical species, while also acting as a barrier for the movement of species between western and eastern Mexico. The SMO houses 57 species of amphibians and 160 species of reptiles, representing 19.4% of the total number of amphibian and reptile species found in Mexico. Ten amphibian and thirteen reptile species are endemic to the SMO, and >50% of amphibian and reptile species found in the SMO are endemic to Mexico. Five amphibian species and three reptile species are listed in a category of conservation concern in the IUCN Red List. Specific threats include habitat loss, logging, mining, pollution, and climate change. The SMO shares a significant percentage of its species with neighboring provinces. In the cluster analysis for amphibians, a group comprising the SMO, the Pacific Lowlands, and the Chihuahuan Desert is linked to the Transvolcanic Belt. For reptiles, two distinct groups emerge: one including the SMO and the Chihuahuan Desert and one including the Transvolcanic Belt and the Pacific Lowlands. In conclusion, the rich biodiversity of the SMO, along with its role as both a corridor and barrier for species distribution, highlights the importance of targeted conservation efforts to mitigate the various threats facing this biogeographically significant province.

## Simple Summary

The Sierra Madre Occidental (SMO) in Mexico is a biologically rich region, home to 57 amphibian species and 160 reptile species, many of which are unique to the area. These species are crucial to the region’s biodiversity, with over half being endemic to Mexico. However, the SMO faces several threats, including habitat loss from logging, mining, and climate change, putting many species at risk. Five amphibians and three reptiles from the region are listed as vulnerable or endangered. The SMO also shares many species with neighboring provinces, underlining the importance of coordinated conservation efforts across these regions. The Mexican government has established protected areas within the SMO, but additional measures are needed to ensure the survival of these species. Protecting the unique wildlife of the SMO is essential for maintaining the region’s ecological balance and contributing to global biodiversity conservation efforts.

## 1. Introduction

The Sierra Madre Occidental (SMO) is a transitional biogeographic province that functions as a corridor for the expansion of subtropical species to the north and west, as well as an important barrier preventing the dispersal of eastern species to the Pacific Coast and of western species to temperate and semiarid regions [1,2,3,4,5,6]. Thus, some areas of the SMO have high biodiversity due to the meeting of species of the Nearctic and Neotropical regions [7]. The SMO is also an area of a high degree of endemism for a variety of organisms [8]. In addition, the SMO is distinguished by a remarkable heterogeneity of habitats resulting in a rich flora and fauna, with the SMO representing an important center of biological diversity for a variety of taxa, including plants [1,5,9], birds [10], and mammals [1,11]. It is also an important region for amphibians and reptiles. For example, the SMO is a hotspot for herpetofaunal endemism in Mexico [12].

The SMO is not only a region of exceptional biodiversity but also a cultural epicenter for several native North American cultures. The Raramuri (or Tarahumara), the O’óba (or Pima [=Mountain Pima]), the Warijío (or Guarijío), the Ódami (or Tepehuan), Nayeeri (or Cora), and Wixárika (or Huichol), among others, have long inhabited this region, each with unique understandings of their surroundings [13]. These indigenous communities have deeply ingrained practices that reflect their harmonious relationship with nature, forming part of their worldviews. Such practices serve as a testament to their commitment to preserving their natural heritage for future generations [3,14,15,16]. This cultural perspective, intertwined with the ecological significance of the SMO, highlights the comprehensive approach necessary for sustainable conservation efforts in this region.

The remoteness of the rugged mountains in the SMO with their relatively low human population densities and cultural conservatism has allowed the region to retain many of its ecosystems in a rather natural state. However, an increasing human population is placing more demands on the environment [17,18,19]. The principal economic activities for residents of the SMO are based on harvesting from forests and woodlands, as well as largely subsistence agriculture and cattle grazing [13,17,20]. Other major threats include dams, logging, firewood cutting, charcoal-making, clearing for agriculture, mining, urbanization, and replacement of the forests with buffelgrass (*Pennisetum ciliare*) at lower elevations in the southern part of the region [14,17]. Mining has devastating effects on the amphibian and reptile species of the SMO. For example, in the municipality of Ocampo, Chihuahua, entire hills have been eliminated for the extraction of gold and silver, including the type locality of *Istmura sierraoccidentalis*, a salamander endemic to the SMO, known only from three populations, one of which is considered extirpated already (Ocampo, Chihuahua) [21].

Given the increasing pressures from human activities such as mining, deforestation, and land-use change, and their documented impacts on biodiversity, it is essential to establish a reliable and up-to-date inventory of the amphibian and reptile species of the Sierra Madre Occidental (SMO). This study has three specific aims: (1) compile a comprehensive and taxonomically updated checklist of amphibians and reptiles occurring within the SMO; (2) assess the conservation status and known distributions of these species, including levels of endemism and potential threats; and (3) examine the biogeographic affinities of the herpetofauna in relation to neighboring regions, contributing to a better understanding of species turnover and habitat connectivity. By achieving these aims, we seek to inform and support more targeted and culturally inclusive conservation strategies in this biologically and culturally significant region.

## 2. Materials and Methods

### 2.1. Physiographic Characteristics

**Figure 1:**
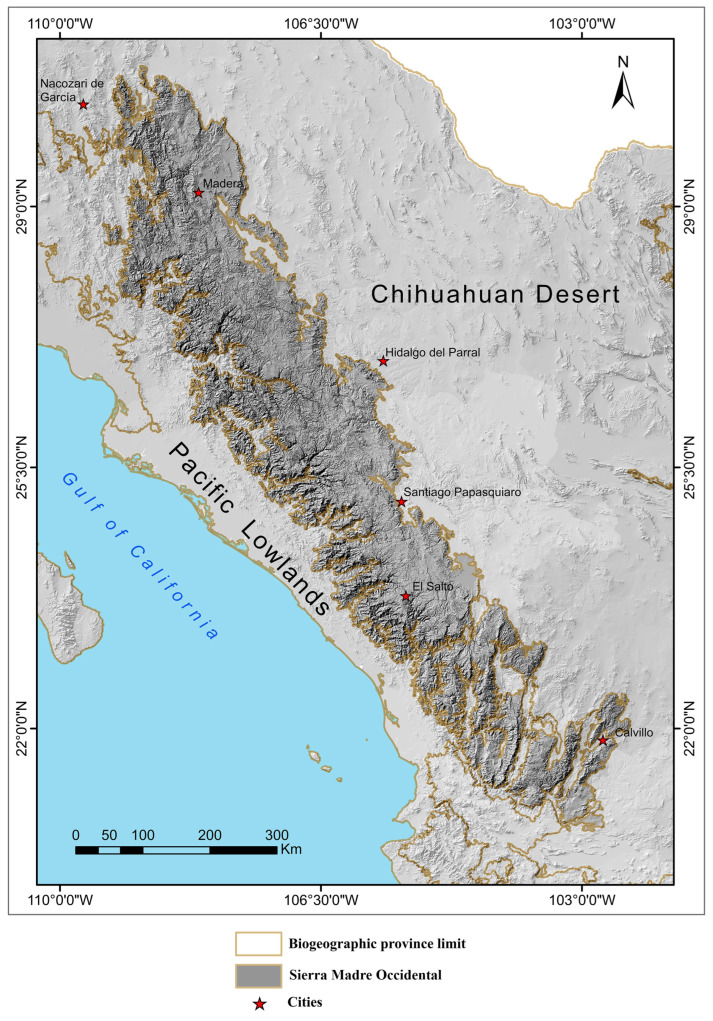
Topography map of the Sierra Madre Occidental biogeographic province of Mexico [22].

The SMO is located in western Mexico and covers 171,195 km^2^ between 20.62° and 30.89° N and 102.35° and 109.43° W, WGS 84 (Figure 1). It occupies parts of the states of Sonora, Chihuahua, Sinaloa, Durango, Zacatecas, Nayarit, and Jalisco. This major mountain range runs parallel to the Pacific Lowlands province and is the longest and most continuous mountain range in Mexico. The SMO is a very rugged area formed mainly of mountains, plateaus, canyons, and valleys. The highest (>3300 m) and lowest (<200 m) elevations of the SMO indicate that the topography of this biogeographic province is highly heterogeneous (Figure 1; [4]). It is also home to the most ecologically and culturally important rivers in northern Mexico [14].

**Figure 2:**
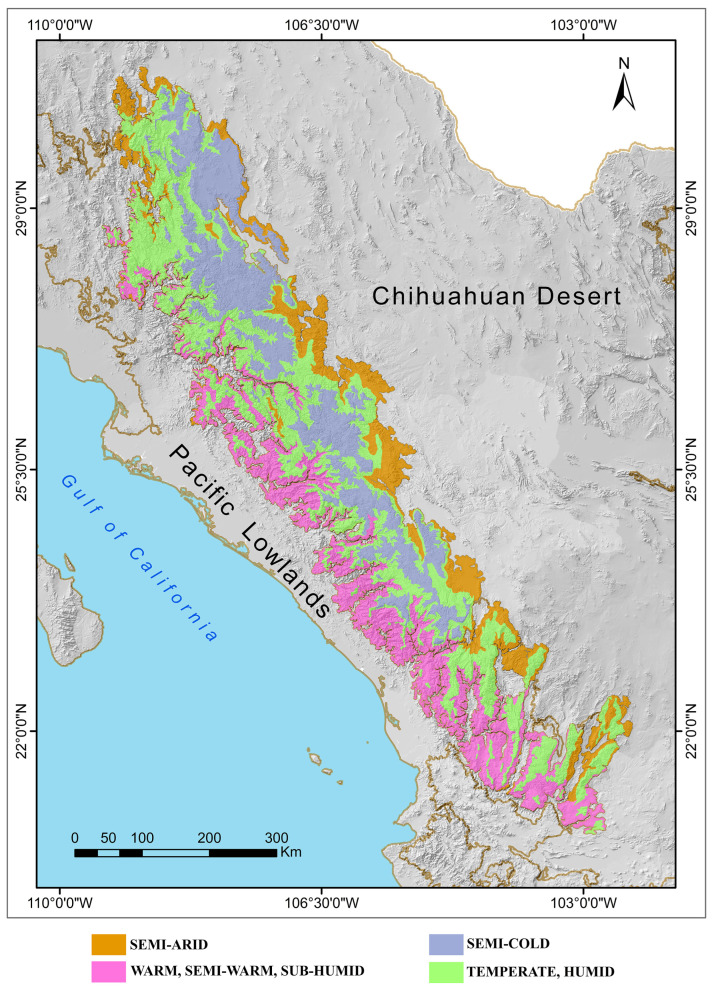
Climate map of the Sierra Madre Occidental biogeographic province of Mexico [23].

The dominant climates in the SMO are semi-cold humid at the highest elevations (precipitation: 500 to 600 mm per year, mean annual temperature: 10 to 12 °C); temperate subhumid at mid-elevations (precipitation: 500 to 600 mm per year, mean annual temperature: 14 °C); semi-warm subhumid in the canyon region contiguous to the Pacific Lowlands province (precipitation: 800 to 1400 mm per year, mean annual temperature: 20 to 24 °C); and a small portion with a warm subhumid climate in the lowlands of the mountains that also form part of the Pacific Lowlands province (precipitation: 700 and 1000 mm per year, mean annual temperature: 24 °C) (Figure 2; [2]).

**Figure 3:**
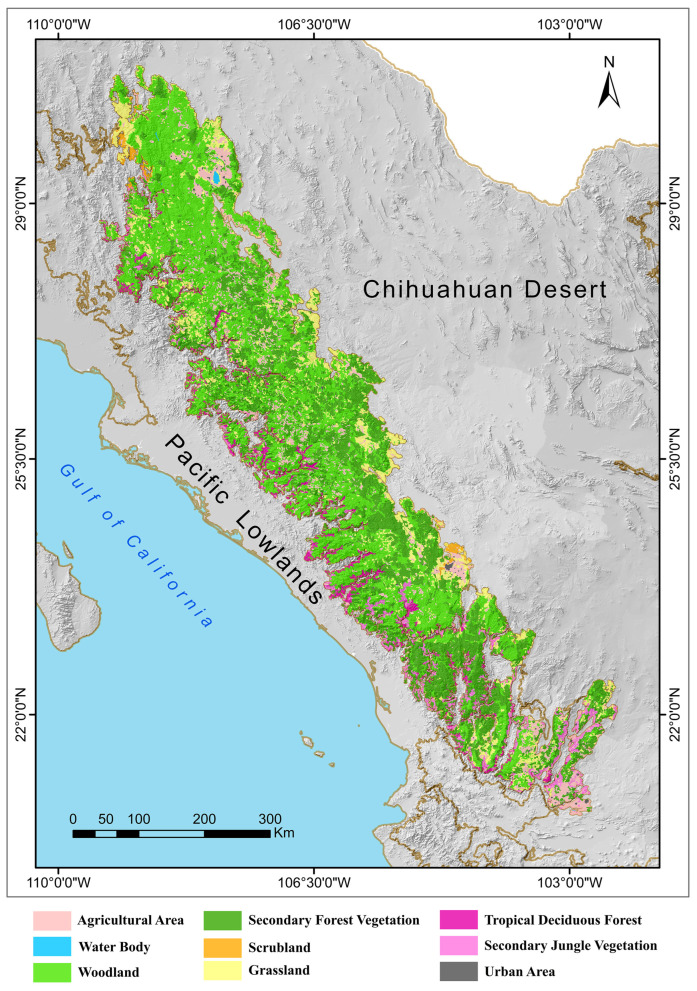
Vegetation map of the Sierra Madre Occidental biogeographic province of Mexico [24].

There are four main types of vegetation in the SMO: Tropical Deciduous Forest; Oak Forest; Pine–Oak Forest; and Mixed Coniferous Forest (Figure 3; see [9]). The vegetation types of the SMO are arranged in longitudinal strips that coincide with the different altitudinal intervals that occur from the lowlands of the western slope to the summit of the Continental Divide in its highest parts. On the border with the Chihuahuan Desert and where the altitudinal variation is not so abrupt, these strips are wider [24].

### 2.2. Methodology

We compiled a list of the species of amphibians and reptiles of the SMO biogeographic province using the species lists of the Mexican states (Sonora, Chihuahua, Sinaloa, Durango, Zacatecas, Nayarit, and Jalisco) that contribute to the SMO provided by [25] and updated with [26]. We confirmed records using VertNet and GBIF, along with other sources, and found no inconsistencies with the published state lists provided by [25], which were compiled by regional experts and peer-reviewed. These lists incorporate data from VertNet, GBIF, museum collections, literature reviews, and fieldwork, further supporting their reliability. We follow the Amphibian Species of the World (https://amphibiansoftheworld.amnh.org/index.php, accessed on 17 March 2025) [27] and AmphibiaWeb (http://amphibiaweb.org, accessed on 17 March 2025) [28] for amphibian names and Reptile Database (http://www.reptile-database.org, accessed on 17 March 2025) [29] for reptile names. We also generated species lists for the amphibians and reptiles of the neighboring provinces (Transvolcanic Belt, Chihuahuan Desert, and Pacific Lowlands) using similar methods (see [26,30,31,32]). We used the definition of the SMO and neighboring biogeographic provinces of [6,33,34,35]. In addition, we recorded the conservation status and population trends of each species using three sources: (1) the International Union for Conservation of Nature’s (IUCN) Red List version 2024-2 (https://www.iucnredlist.org/, accessed on 14 March 2025), which offers regularly updated, globally standardized assessments of extinction risk and population trends [21]; (2) the official species-at-risk list published by the Mexican government through SEMARNAT [36], which provides national-level risk categories, although its most recent update was in 2010 and is therefore considered outdated; and (3) Environmental Vulnerability Scores (EVS), which provide a qualitative assessment of species vulnerability based on geographic distribution, ecological specialization, and anthropogenic threats [37,38]. It is important to note that EVS assessments are expert-driven and incorporate subjective criteria, and while useful for identifying potential conservation concerns, they are not regularly updated and do not track population trends over time. While the IUCN Red List offers the most current and dynamic conservation data (as of March 2025), the SEMARNAT list and EVS do not allow for robust temporal comparisons due to their static or outdated nature. Therefore, our analysis focuses on current conservation status rather than long-term trends, given the limitations of the available national and regional datasets.

We used hierarchical clustering analyses based on Jaccard’s Similarity Coefficients for Binary Data as the distance metric with single linkages methods (nearest neighbor) to generate clusters of the amphibian and reptile species of the SMO and the neighboring biogeographic provinces. Cluster analyses were performed using Systat 13.2 (Systat Software Inc., San Jose, CA, USA).

## 3. Results and Discussion

### 3.1. Species Richness

**Figure 4:**
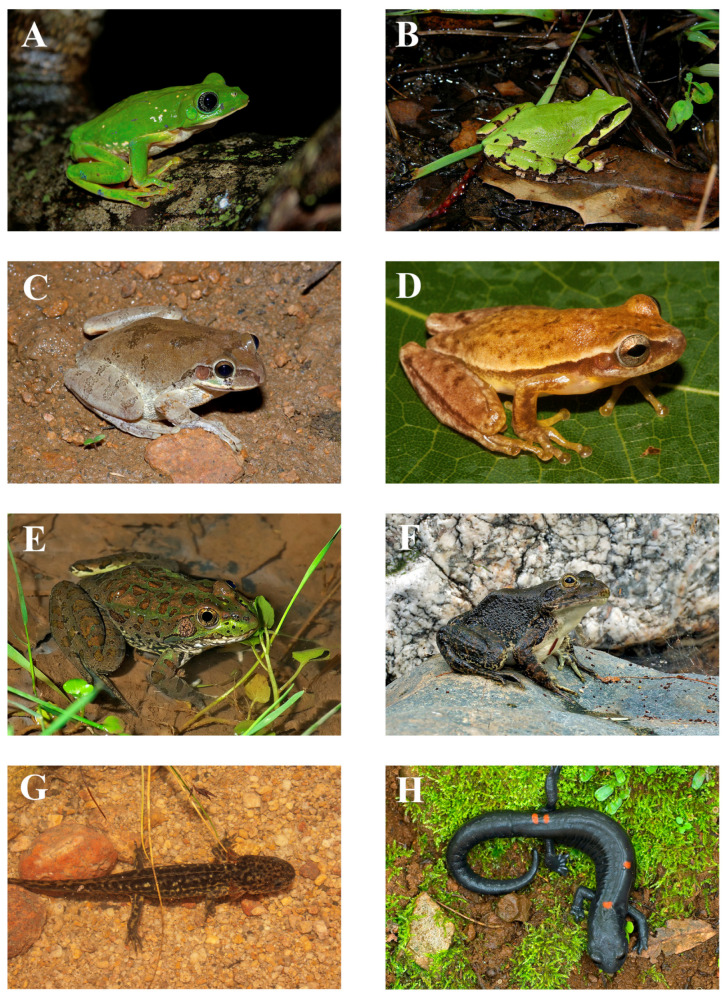
Photos of some of the amphibian species native to the Sierra Madre Occidental: (**A**) *Agalychnis dacnicolor*, El Monteón, Nayarit; (**B**) *Dryophytes wrightorum*; (**C**) *Smilisca baudinii*, Sonora; (**D**) *Tlalocohyla smithii*, Pihuamo, Jalisco; (**E**) *Rana chiricahuensis*, Sonora; (**F**) *Rana tarahumarae*, Sonora; (**G**) *Ambystoma rosaceum*, Sierra Los Huicholes, Jalisco; (**H**) *Isthmura sierraoccidentalis*, Yécora, Sonora. Photos (**A**,**D**,**G**) by Iván Ahumada Carrillo; Photo (**B**) by William Wells; Photos (**C**,**E**,**F**,**H**) by Erick Enderson.

**Figure 5:**
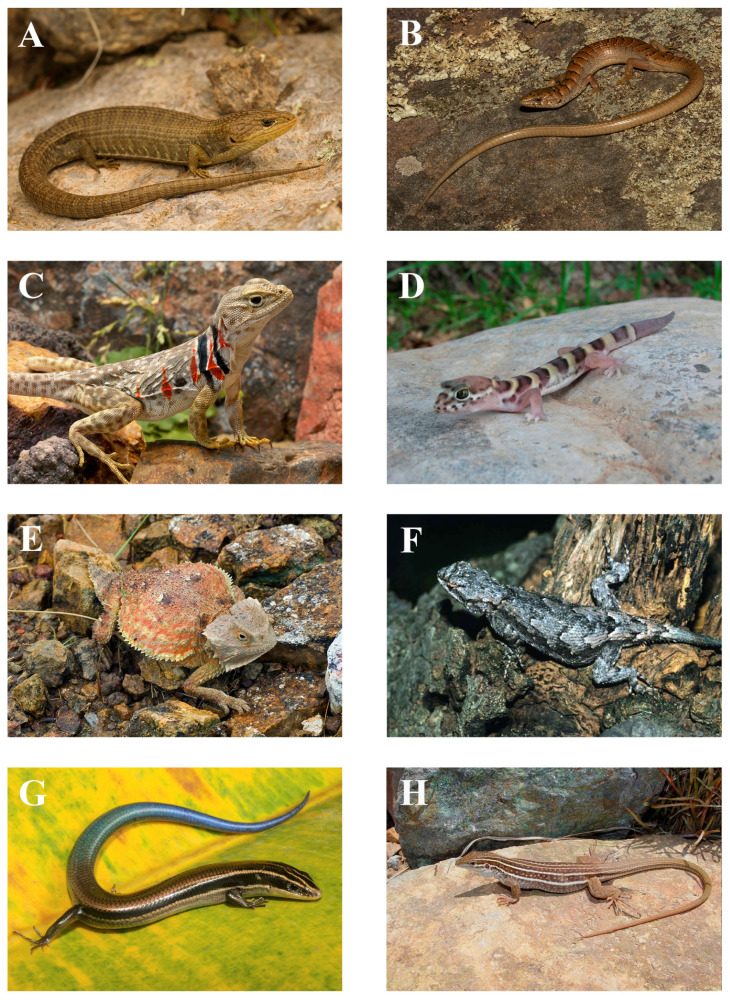
Photos of some of the lizard species native to the Sierra Madre Occidental: (**A**) *Barisia levicollis*, Sierra del Nido, Chihuahua; (**B**) *Elgaria kingii*, Bosque La Primavera, Jalisco; (**C**) *Crotaphytus nebrius*; (**D**) *Coleonyx fasciatus*, Álamos, Sonora; (**E**) *Phrynosoma ditmarsi*, Sonora; (**F**) *Sceloporus lemosespinali*, Estación San Rafael, Urique, Chihuahua; (**G**) *Plestiodon callicephalus*, Zapopán, Jalisco; (**H**) *Aspidoscelis sonorae*. Photo (**A**) by Marisa Ishimatsu; Photos (**B**,**G**) by Iván Ahumada Carrillo; Photo (**C**) by Barney Oldfield; Photo (**D**) by Matt Cage; Photo (**E**) by Erick Enderson; Photo (**F**) by Peter Heimes; Photo (**H**) by Thomas C. Brennan.

**Figure 6:**
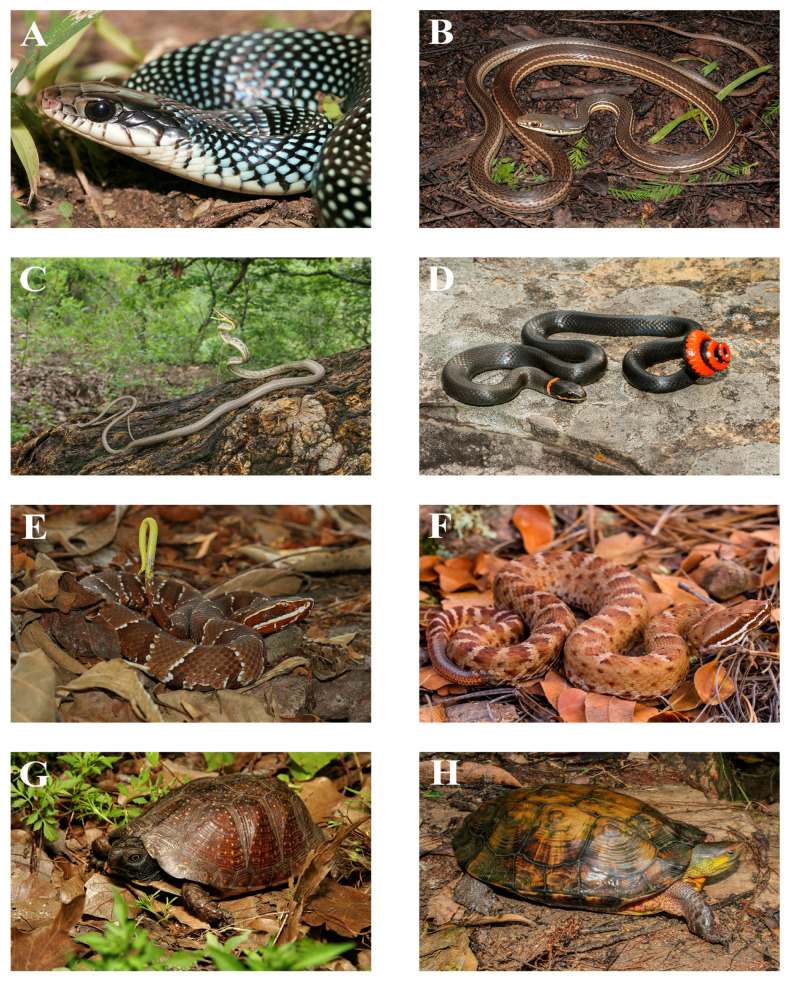
Photos of some of the snake and turtle species native to the Sierra Madre Occidental: (**A**) *Drymobious margaritiferus*, Álamos, Sonora; (**B**) *Masticophis bilineatus*, Mezquital del Oro, Zacatecas; (**C**) *Oxybelis microphthalmus*, Sierra de Quila, Jalisco; (**D**) *Diadophis punctatus*, Tapalpa, Jalisco; (**E**) *Agkistrodom bilineatus*, Zapopán, Jalisco; (**F**) *Crotalus willardi*, Sierra del Nido, Chihuahua; (**G**) *Terrapene nelsoni*, Yécora, Sonora; (**H**) *Rhinoclemmys pulcherrima*, Sierra de Manantlán, Jalisco. Photos (**A**,**G**) by Young Cage; Photos (**B**–**D**,**E**,**H**) by Iván Ahumada Carrillo; Photo (**F**) by Marisa Ishimatsu.

The SMO houses 217 native species of amphibians and reptiles—57 amphibians and 160 reptiles—representing 34 families, 11 of which are amphibians (9 anurans and 2 salamanders) and 23 of which are reptiles (11 lizards, 7 snakes, and 3 turtles), and 66 genera (19 amphibians and 47 reptiles) (Table 1; see Figure 4, Figure 5 and Figure 6 for some of the amphibian and reptile species native to the SMO). Compared to the total number of families and species present in Mexico, these numbers are relatively low. According to [26], the total number of native amphibian and reptile species in Mexico is 1399 (435 amphibians and 964 reptiles), from 55 families (16 amphibians and 39 reptiles) and 210 genera (55 amphibians and 155 reptiles). These numbers are similar to those reported by [39]. Therefore, the SMO harbors around 62% of the families, 43% of the genera, and 16% of the species of amphibians and reptiles in Mexico. For amphibians, the SMO is home to around 69% of the families, 34% of the genera, and 13% of the species in Mexico, and for reptiles, 59% of the families, 30% of the genera, and 17% of the species in Mexico. In addition, four species have been introduced to the SMO: the American Bullfrog (*Rana catesbeiana*), the Stump-toed Gecko (*Gehyra mutilata*), the Brahminy Blind Snake (*Indotyphlops braminus*), and the Yellow-bellied Slider Turtle (*Trachemys scripta*) (Table 1).

### 3.2. Endemism

Ten species of amphibians (18%) are endemic to the SMO (Table 1). A further 26 species (46%) of amphibians that occur in the SMO but that also occur in other biogeographic provinces are endemic to Mexico, for a total of 36 species (63%) found in the SMO that are endemic to Mexico. Thirteen species of reptiles (8%) are endemic to the SMO, with another 73 species (40%) that occur in the SMO endemic to Mexico but also occurring in other biogeographic provinces, making a total of 86 species (54%) that are endemic to Mexico. Our results confirm that the SMO is an endemic-rich biogeographic region for amphibians and reptiles [12], with >50% of the species of amphibians and reptiles being Mexican endemics. In addition to the high proportion of endemic species, the SMO ranks fifth in the number of Mexican endemic species among the Mexican provinces [12]. Therefore, this province is among the more important areas of endemism in Mexico that are at risk of continued and accelerating habitat loss and conversion by expanding human populations [17,18,19].

### 3.3. Comparison with Neighboring Provinces

**Figure 7:**
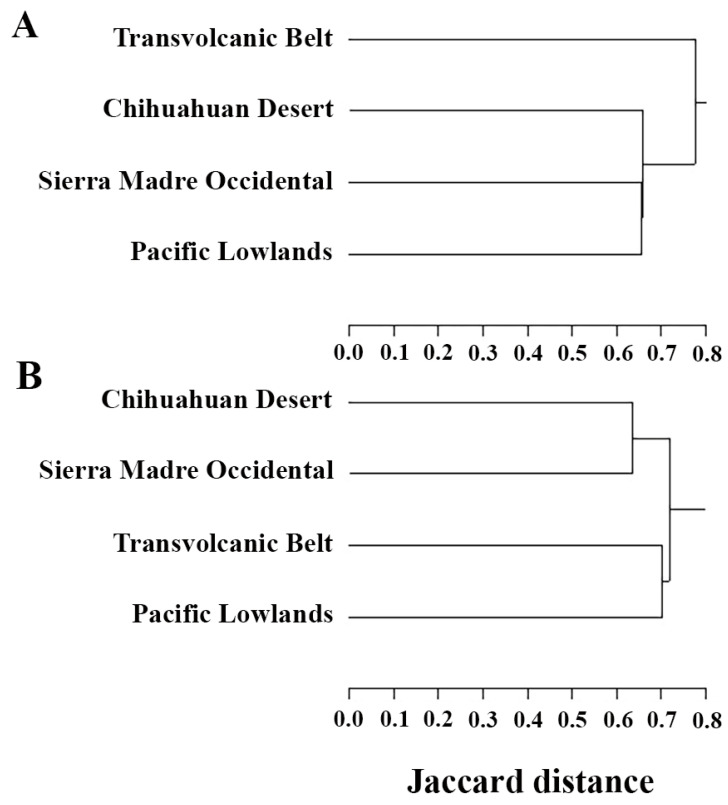
Cluster trees for amphibians (**A**) of the Sierra Madre Occidental and its neighboring biogeographic provinces and (**B**) cluster trees for reptiles of the Sierra Madre Occidental and its neighboring biogeographic provinces.

The SMO shares 60.4% of its species with the Transvolcanic Belt, 58.1% with the Chihuahuan Desert, and 54.8% with the Pacific Lowlands (Table 2). For amphibians, the SMO shares 63.2% of its native species with the Transvolcanic Belt, 59.6% with the Pacific Lowlands, and 49.1% with the Chihuahuan Desert (Table 2). The cluster analysis for amphibians in the SMO and neighboring provinces reveals a distinct group comprising the SMO, the Pacific Lowlands, and the Chihuahuan Desert, which is linked to the Transvolcanic Belt (Figure 7A). The SMO shares 59.4% of its native reptile species with the Transvolcanic Belt, 61.3% with the Chihuahuan Desert, and 53.1% with the Pacific Lowlands (Table 2). In the cluster analysis for reptiles, two distinct groups emerge: one includes the SMO and the Chihuahuan Desert, and the other includes the Transvolcanic Belt and the Pacific Lowlands (Figure 7B). The number of shared species between the SMO and its neighboring provinces likely arises because the SMO is a transitional biogeographic province where Neotropical and Nearctic species mix, unlike the Pacific Lowlands where Neotropical species predominate and the Chihuahuan Desert where Nearctic species predominate. When comparing the similarities of the entire herpetofauna of biogeographic provinces within states, the SMO is frequently most similar to the Transvolcanic Belt [30], which is in line with our results for total herpetofauna.

### 3.4. Conservation Status

**Figure 8:**
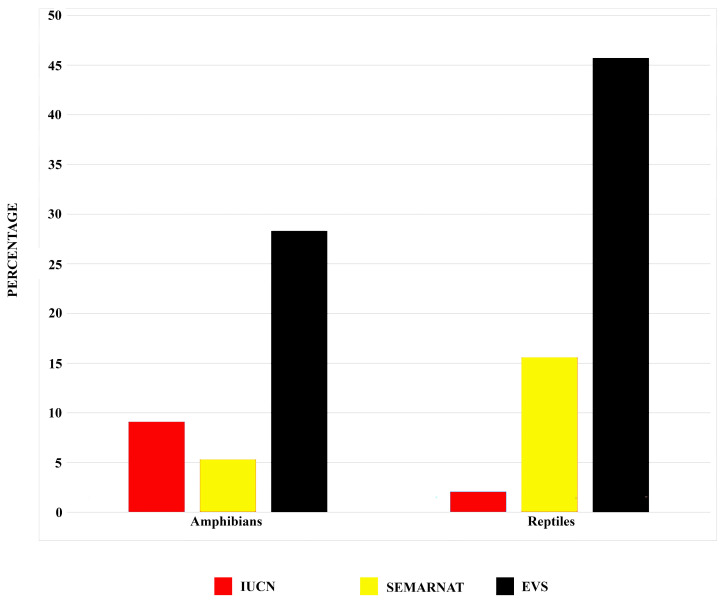
Percentage of amphibian and reptile species with conservation concern status (IUCN) [21], categorized as threatened (A) or in danger of extinction (P) by the Mexican government (SEMARNAT) [36], or deemed to have a high environmental vulnerability score (EVS) [37,38], for the Sierra Madre Occidental biogeographic provinces of Mexico.

Five species of amphibians and three species of reptiles that occur in the SMO are IUCN [21] listed as Vulnerable, Endangered, or Critically Endangered; 28 (3 amphibians and 25 reptiles) are categorized as threatened (A) or in danger of extinction (P) by the Mexican government (SEMARNAT) [36]; and 84 (15 amphibians and 69 reptiles) are categorized as high risk by the Environmental Vulnerability Score (EVS) (Table 3; Figure 8). These results indicate that the different ways of assessing conservation status (e.g., IUCN, SEMARNAT, and EVS) give different perspectives on the conservation status of the species of the SMO. In particular, it appears the IUCN may be underestimating the number of species in the SMO that are at risk. In part, the disconnect between the IUCN and SEMARNAT, and especially between the IUCN and EVS, likely reflects the global nature of the IUCN assessment rather than the more local or regional knowledge used in SEMARNAT and EVS assessments.

While species of conservation concern are found across the taxa found in the SMO, some families appear to be of particular concern, as evidenced by the relatively high frequency of species in those taxa that are in a category of concern in the IUCN, SEMARNAT, or the EVS. In amphibians, Eleutherodactylidae, Ranidae, and Ambystomatidae are families of particular concern (Table 1 and Table 3). For reptiles, Helodermatidae, Natricidae, Viperidae, and Kinosternidae are families warranting concern (Table 1 and Table 3).

All eight species listed in categories of conservations concern by the IUCN are facing habitat loss through agriculture, urbanization, subsisting or commercial logging, mining, and/or industry as a threat to their existence [21]. Indeed, habitat loss threatens several species in the mountainous regions of Mexico (e.g., [40]). Anthropogenic threats to endemic Mexican amphibians are relatively high in parts of the SMO [41] and include forest loss due to lumber extraction and the disruption of the fire regime [19,42,43]. In addition, the forests of the SMO are likely to be particularly affected by climate change [44]. Urbanization is also a threat to much of Mexico’s biodiversity [45]. The Sierra Madre Occidental (SMO) appears to be climatically suitable for the amphibian chytrid fungus *Batrachochytrium dendrobatidis* (*Bd*), as ecological niche models developed by [46] identified the SMO pine–oak forest as one of the regions in the Neotropics with the highest predicted environmental suitability for *Bd* (occupancy index ≥ 0.7). These models, based on a range of temperature and precipitation conditions from known *Bd* localities across the New World, demonstrated that *Bd* can persist in diverse climates, including areas with high annual precipitation and elevation, conditions characteristic of the SMO. Furthermore, the strong predictive power of the models across both the New and Old Worlds supports their robustness in identifying climatically suitable habitats for *Bd*, reinforcing the conclusion that the SMO provides a favorable environmental envelope for the pathogen.

The Mexican government, through the National Commission of Natural Protected Areas (CONANP), has established ten protected natural areas in the SMO, covering 17.6% of the SMO. These areas fall into four protection categories: Biosphere Reserve (represents the diversity of the country’s ecosystems and the representativeness in terms of biological diversity and the presence of endemic, threatened, or endangered species); National Park (established in sites with ecosystems that mainly have scenic beauty, historical, scientific, educational, and recreational value, that conserve special flora and fauna, and, above all, that are suitable for tourism development); natural resource protection areas (includes any area dedicated to the preservation and protection of soils, watersheds, waters, and natural resources of forest lands); and flora and fauna protection areas (where the main focus is species conservation) [47]. Additionally, the SMO includes 32 of the 152 Priority Terrestrial Regions (PTRs) of Mexico established by the National Commission for the Understanding and Use of Biodiversity (CONABIO) [5]. Among other objectives, CONABIO promotes biological research in these PTR, which in many cases results in conservation proposals for amphibian and reptile species and increases the understanding of the situation of the populations of these species in these PTR. However, additional protected areas are needed to ensure connectivity among existing protected areas [48].

In addition to the presence of threatened species, it is important to consider the potential impact of invasive amphibians and reptiles on native and endemic herpetofauna in the SMO. At least four non-native species have been documented in the region: the American Bullfrog (*Rana catesbeiana*), the Stump-toed Gecko (*Gehyra mutilata*), the Brahminy Blindsnake (*Indotyphlops braminus*), and the Yellow-bellied Slider (*Trachemys scripta*). While direct evidence of their ecological effects within the SMO remains limited, research from other regions suggests they may pose significant threats to native biodiversity. *Rana catesbeiana* is a large, generalist predator known to prey on a wide range of native species, including amphibians, reptiles, birds, and invertebrates. Its presence is associated with declines in native frog populations due to predation, competition, disease transmission, particularly chytridiomycosis, and reproductive interference. Its success is often supported by anthropogenic changes such as permanent water bodies and the introduction of non-native fish [49]. *Gehyra mutilata* is an opportunistic, nocturnal gecko with a broad diet mainly composed of arthropods, which may enable it to outcompete native gecko species. In addition, studies have shown that it may possess physiological advantages that enhance its ability to invade and establish in novel environments, particularly disturbed or urbanized areas [50]. *Indotyphlops braminus*, a parthenogenetic species introduced through soil and potted plants, likely has limited direct interaction with native vertebrates but could influence local invertebrate populations. Its ability to occupy the same microhabitats as native fossorial snakes raises concerns about potential competition, especially in ecosystems where space and prey are limiting factors [51,52]. *Trachemys scripta*, similarly, poses significant risks to native aquatic species, especially turtles, by aggressively competing for basking sites and food resources. Studies from Europe demonstrate that its presence leads to behavioral changes, reduced reproductive success, and increased mortality in native terrapin populations. The lack of evolved antipredator responses among native amphibians further exacerbates its predatory impact [53]. Although empirical studies on the impacts of these species in the SMO are lacking, the ecological risks documented elsewhere underscore the importance of monitoring their spread and investigating their potential to disrupt native communities.

## 4. Conclusions

The SMO is a biogeographic province of considerable species richness, housing 57 native amphibian species and 160 native reptile species. Thirty-six of the 57 native amphibian species of the SMO are endemic to Mexico, with 11 of them endemic to the SMO. Eighty-six of the 160 native reptile species are endemic to Mexico, with 10 of them endemic to the SMO. The SMO shares just over half its herpetofaunal species with its neighboring province, the Transvolcanic Belt, Chihuahuan Desert, and Pacific Lowlands. This sharing of species highlights the importance of regional conservation efforts and collaboration among different biogeographic provinces to protect biodiversity effectively. Moreover, the cluster analysis for amphibians reveals distinct groupings of provinces, with the SMO showing closer associations with the Pacific Lowlands and the Chihuahuan Desert than with the Transvolcanic Belt, and in the cluster analysis for reptiles, two distinct groups emerge: one includes the SMO and the Chihuahuan Desert, and the other the Transvolcanic Belt and the Pacific Lowlands. This clustering pattern, along with the number of shared species between the SMO and its neighboring provinces, suggests the need for coordinated conservation strategies across these biogeographic provinces. The SMO includes 23 species of conservation concern, due to threats such as habitat loss, pollution, and climate change, which are particularly detrimental to species already at risk. Conservation efforts by the Mexican government, including the establishment of protected natural areas, are crucial for safeguarding these species and their habitats. Overall, our results suggest the SMO is a crucial region for Mexican amphibians and reptiles, with a higher level of endemism, and highlight the need for conservation efforts to protect its unique biodiversity.

## Figures and Tables

**Table 1:** Native amphibians and reptiles of the Sierra Madre Occidental biogeographic province with distributional and conservation status. IUCN Status (DD = Data Deficient; LC = Least Concern; NT = Near Threatened; VU = Vulnerable; EN = Endangered; CE = Critically Endangered; NE = not Evaluated; Trend (↑)—Increasing; (↓)—Decreasing; (=)—Stable; (?)—Unknown), according to the IUCN Red List [21], Environmental Vulnerability Score (EVS—the higher the score the greater the vulnerability: low (L) vulnerability species (EVS of 3–9); medium (M) vulnerability species (EVS of 10–13); and high (H) vulnerability species (EVS of 14–20) from [37,38], and Mx indicates category of risk in Mexico according to SEMARNAT [36]: P (in danger of extinction); A (threatened); Pr (subject to special protection); NL (not listed). Global = Global Distribution: 0 = Endemic to the Sierra Madre Occidental; 1 = Endemic to Mexico; 2 = Shared between the US and Mexico; 3 = widely distributed from Mexico to Central America; 4 = widely distributed from Canada or the US to Central or South America. Total refers to the number of Biogeographic Provinces that the species inhabits: Endemic (EN) = 1 province, so it is endemic to the Sierra Madre Occidental; introduced is a non-native species; (IN) = Introduced to the Sierra Madre Occidental.

	IUCN	EVS	Mx	Global	Total
**Class Amphibia**					
**Order Anura**					
**Bufonidae**					
*Anaxyrus cognatus* (Say, 1822)	LC (↓)	L (9)	NL	2	5
*Anaxyrus compactilis* (Wiegmann, 1833)	LC (?)	H (14)	NL	1	6
*Anaxyrus debilis* (Girard, 1854)	LC (=)	L (7)	Pr	2	5
*Anaxyrus kelloggi* (Taylor, 1938)	LC (=)	H (14)	NL	1	3
*Anaxyrus mexicanus* (Brocchi, 1879)	LC (↓)	M (13)	NL	0	EN
*Anaxyrus punctatus* (Baird & Girard, 1852)	LC (−)	L (5)	NL	2	10
*Anaxyrus woodhousii* (Girard, 1854)	LC (=)	M (10)	NL	2	4
*Incilius alvarius* (Girard, 1859)	LC (=)	M (11)	NL	2	5
*Incilius marmoreus* (Wiegmann, 1833)	LC (=)	M (11)	NL	1	8
*Incilius mazatlanensis* (Taylor, 1940)	LC (=)	M (12)	NL	1	5
*Incilius mccoy* Santos-Barrera & Flores-Villela, 2011	LC (=)	H (14)	NL	0	EN
*Incilius occidentalis* (Camerano, 1879)	LC (=)	M (11)	NL	1	7
*Rhinella horribilis* (Wiegmann, 1833)	LC (↑)	L (3)	NL	4	12
**Craugastoridae**					
*Craugastor augusti* (Dugès, 1879)	LC (=)	L (8)	NL	2	9
*Craugastor hobartsmithi* (Taylor, 1937)	LC (=)	H (15)	NL	1	5
*Craugastor occidentalis* (Taylor, 1941)	LC (=)	M (13)	NL	1	6
*Craugastor rubinus* Jameson, Streicher, Manuelli, Head, & Smith, 2022	NE	NE	NL	0	EN
*Craugastor tarahumaraensis* (Taylor, 1940)	LC (?)	H (17)	Pr	0	EN
*Craugastor vocalis* (Taylor, 1940)	LC (↓)	M (13)	NL	1	5
**Eleutherodactylidae**					
*Eleutherodactylus interorbitalis* (Langebartel & Shannon, 1956)	LC (=)	H (15)	Pr	1	2
*Eleutherodactylus jamesdixoni* Devitt, Tseng, Taylor-Adair, Koganti, Timugura, Cannatella, 2023	NE	NE	NL	1	4
*Eleutherodactylus pallidus* (Duellman, 1958)	LC (=)	H (17)	Pr	1	3
*Eleutherodactylus saxatilis* (Webb, 1962)	NT (=)	H (17)	NL	0	EN
*Eleutherodactylus teretistes* (Duellman, 1958)	VU (?)	H (16)	NL	1	3
*Eleutherodactylus wixarika* Reyes-Velasco, Ahumada-Carrillo, Burkhardt, & Devitt, 2015	EN (↓)	H (18)	NL	0	EN
**Hylidae**					
*Agalychnis dacnicolor* (Cope, 1864)	LC (↓)	M (11)	NL	1	5
*Dryophytes arenicolor* (Cope, 1886)	LC (=)	L (7)	NL	2	8
*Dryophytes eximius* (Baird, 1854)	LC (=)	M (10)	NL	1	7
*Dryophytes wrightorum* (Taylor, 1938)	LC (=)	L (9)	NL	2	2
*Exerodonta smaragdina* (Taylor, 1940)	LC (↓)	M (12)	Pr	1	6
*Sarcohyla hapsa* Campbell et al., 2018	LC (?)	NE	NL	1	5
*Smilisca baudinii* (Duméril & Bibron, 1841)	LC (=)	L (3)	NL	4	11
*Smilisca fodiens* (Boulenger, 1882)	LC (=)	L (8)	NL	2	7
*Tlalocohyla smithii* (Boulenger, 1902)	LC (=)	M (11)	NL	1	6
**Leptodactylidae**					
*Leptodactylus melanonotus* (Hallowell)	LC (=)	L (6)	NL	3	11
**Microhylidae**					
*Gastrophryne mazatlanensis* (Taylor, 1943)	LC (?)	L (8)	NL	2	3
*Hypopachus ustus* (Cope, 1866)	LC (=)	L (7)	Pr	3	8
*Hypopachus variolosus* (Cope, 1866)	LC (=)	L (4)	NL	4	11
**Ranidae**					
*Rana berlandieri* Baird, 1854	LC (=)	L (7)	Pr	2	9
*Rana catesbeiana* Shaw, 1802					IN
*Rana chiricahuensis* Platz & Mecham, 1979	VU (↓)	M (11)	A	2	2
*Rana forreri* Boulenger, 1883	LC (=)	L (3)	Pr	3	8
*Rana lemosespinali* Smith & Chiszar, 2003	DD (?)	H (14)	NL	0	EN
*Rana magnaocularis* Frost & Bagnara, 1976	LC (?)	M (12)	NL	1	6
*Rana megapoda* Taylor, 1942	NT (↓)	H (14)	Pr	1	5
*Rana montezumae* Baird, 1854	LC (↓)	M (13)	Pr	1	6
*Rana neovolcanica* Hillis & Frost, 1985	LC (=)	M (13)	A	1	6
*Rana psilonota* Webb, 2001	LC (?)	H (14)	NL	1	4
*Rana pustulosa* Boulenger, 1883	LC (=)	L (9)	Pr	1	5
*Rana tarahumarae* Boulenger, 1917	VU (↓)	L (8)	NL	2	1
*Rana yavapaiensis* Platz & Frost, 1984	LC (↓)	M (12)	Pr	2	3
**Scaphiopodidae**					
*Scaphiopus couchi* Baird, 1854	LC (=)	L (3)	NL	2	10
*Spea multiplicata* (Cope, 1863)	LC (=)	L (6)	NL	2	9
**Order Caudata**					
**Ambystomatidae**					
*Ambystoma rosaceum* Taylor, 1941	LC (?)	H (14)	Pr	0	EN
*Ambystoma silvense* Webb, 2004	DD (?)	H (14)	NL	0	EN
*Ambystoma velasci* (Dugès, 1888)	LC (?)	M (10)	Pr	1	6
**Plethodontidae**					
*Isthmura belli* (Gray, 1850)	LC (?)	M (12)	A	1	5
*Isthmura sierraoccidentalis* (Lowe, Jones & Wright, 1968)	VU (?)	NE	NL	0	EN
**Class Reptilia**					
**Order Squamata**					
**Suborder Lacertilia**					
**Anguidae**					
*Barisia ciliaris* (Smith, 1942)	NE	H (15)	NL	1	3
*Barisia levicollis* Stejneger, 1890	DD (?)	H (15)	Pr	0	EN
*Elgaria kingii* Gray, 1838	LC (=)	M (10)	Pr	2	5
*Gerrhonotus infernalis* Baird, 1859	LC (=)	M (13)	NL	2	4
*Gerrhonotus liocephalus* Wiegmann, 1828	LC (=)	L (6)	Pr	4	9
**Anolidae**					
*Anolis nebulosus* (Wiegmann, 1834)	LC (=)	M (13)	NL	1	6
**Crotaphytidae**					
*Crotaphytus collaris* (Say, 1823)	LC (=)	M (13)	A	2	4
*Crotaphytus nebrius* Axtell & Montanucci, 1977	LC (=)	M (12)	NL	2	2
**Eublepharidae**					
*Coleonyx fasciatus* (Boulenger, 1885)	LC (↓)	H (17)	NL	1	2
**Gekkonidae**					
*Gehyra mutilata* (Wiegmann, 1834)					IN
**Helodermatidae**					
*Heloderma exasperatum* Bogert & Martin del Campo, 1956	LC (↓)	NE	NL	1	2
*Heloderma horridum* (Wiegmann, 1829)	LC (↓)	M (11)	A	3	6
*Heloderma suspectum* Cope, 1869	NT (↓)	H (15)	A	2	3
**Iguanidae**					
*Ctenosaura macrolopha* Smith, 1972	LC (↓)	H (19)	NL	1	3
*Ctenosaura pectinata* (Wiegmann, 1834)	LC (↓)	H (15)	A	1	7
**Phrynosomatidae**					
*Cophosaurus texanus* Troschel, 1852	LC (=)	H (14)	A	2	6
*Holbrookia approximans* Baird, 1859	NE	H (14)	NL	1	2
*Holbrookia elegans* Bocourt, 1874	LC (=)	M (13)	NL	2	4
*Phrynosoma cornutum* (Harlan, 1825)	LC (=)	M (11)	NL	2	5
*Phrynosoma ditmarsi* Stejneger, 1906	DD (?)	H (16)	NL	0	EN
*Phrynosoma hernandesi* Girard, 1858	LC (=)	M (13)	NL	2	2
*Phrynosoma orbiculare* (Linnaeus, 1766)	LC (=)	M (12)	A	1	6
*Phrynosoma ornatissimum* (Girard, 1858)	NE	NE	NL	2	2
*Phrynosoma solare* Gray, 1845	LC (=)	H (14)	NL	2	4
*Sceloporus albiventris* Smith, 1939	NE	H (16)	NL	1	3
*Sceloporus asper* Boulenger, 1897	LC (↓)	H (14)	Pr	1	4
*Sceloporus aurantius* Grummer & Bryson, 2014	NE	H (16)	NL	1	2
*Sceloporus brownorum* Smith, Watkins-Colwell, Lemos-Espinal, & Chiszar, 1997	NE	H (15)	NL	0	EN
*Sceloporus bulleri* Boulenger, 1894	LC (=)	H (15)	NL	1	4
*Sceloporus clarkii* Baird & Girard, 1852	LC (=)	M (10)	NL	2	5
*Sceloporus dugesii* Bocourt, 1874	LC (=)	M (13)	NL	1	4
*Sceloporus grammicus* Wiegmann, 1828	LC (=)	L (9)	Pr	2	8
*Sceloporus heterolepis* Boulenger, 1895	LC (?)	H (14)	NL	1	5
*Sceloporus horridus* Wiegmann, 1834	LC (=)	M (11)	NL	1	6
*Sceloporus huichol* Flores-Villela, Smith, Campillo-García, Martínez-Méndez, & Campbell, 2022	NE	NE	NL	1	2
*Sceloporus jarrovii* Cope, 1875	LC (=)	M (11)	NL	2	3
*Sceloporus lemosespinali* Lara-Góngora, 2004	DD (?)	H (16)	NL	0	EN
*Sceloporus melanogaster* Cope, 1885	NE	NE	NL	1	3
*Sceloporus melanorhinus* Bocourt, 1876	LC (=)	L (9)	NL	3	6
*Sceloporus nelsoni* Cochran, 1923	LC (=)	M (13)	NL	1	4
*Sceloporus poinsettii* Baird & Girard, 1852	LC (=)	M (12)	NL	2	4
*Sceloporus scalaris* Wiegmann, 1828	LC (=)	M (12)	NL	1	6
*Sceloporus shannonorum* Langebartel, 1959	DD (?)	H (15)	NL	1	2
*Sceloporus slevini* Smith, 1937	LC (↓)	M (11)	NL	2	2
*Sceloporus spinosus* Weigmann, 1828	LC (=)	M (12)	NL	1	7
*Sceloporus unicanthalis* Smith, 1937	NE	H (16)	NL	1	4
*Sceloporus utiformis* Cope, 1864	LC (=)	H (15)	NL	1	6
*Sceloporus virgatus* Smith, 1938	LC (=)	H (15)	NL	2	1
*Urosaurus bicarinatus* (Duméril, 1856)	LC (=)	M (12)	NL	1	7
*Urosaurus ornatus* (Baird & Girard, 1852)	LC (=)	M (10)	NL	2	5
**Phyllodactylidae**					
*Phyllodactylus saxatilis* Dixon, 1964	NE	NE	NL	1	2
*Phyllodactylus lanei* Smith, 1935	LC (=)	H (15)	NL	1	5
**Scincidae**					
*Plestiodon bilineatus* (Tanner, 1958)	NE	M (13)	NL	0	EN
*Plestiodon callicephalus* (Bocourt, 1879)	LC (=)	M (12)	NL	2	4
*Plestiodon lynxe* (Wiegmann, 1834)	LC (=)	M (10)	Pr	1	6
*Plestiodon multilineatus* (Tanner, 1957)	DD (?)	H (16)	Pr	0	EN
*Plestiodon obsoletus* (Baird & Girard, 1852)	LC (=)	M (11)	NL	2	6
*Plestiodon parviauriculatus* (Taylor, 1933)	DD (?)	H (15)	Pr	1	2
*Plestiodon parvulus* (Taylor, 1933)	DD (?)	H (15)	NL	1	4
**Teiidae**					
*Aspidoscelis costatus* (Cope, 1878)	LC (=)	M (11)	Pr	1	8
*Aspidoscelis exsanguis* (Lowe, 1956)	LC (=)	H (14)	NL	2	2
*Aspidoscelis gularis* (Baird & Girard, 1852)	LC (=)	L (9)	NL	2	6
*Aspidoscelis lineattissimus* (Cope, 1878)	LC (=)	H (14)	Pr	1	5
*Aspidoscelis opatae* (Wright, 1967)	DD (?)	H (16)	NL	0	EN
*Aspidoscelis preopatae* Barley, Reeder, Nieto-Montes de Oca, Cole & Thomson, 2021	NE	NE	NL	0	EN
*Aspidoscelis sonorae* (Lowe & Wright, 1964)	LC (=)	M (13)	NL	2	3
*Aspidoscelis stictogrammus* (Burger, 1950)	LC (=)	H (14)	NL	2	3
**Xantusidae**					
*Xantusia sanchezi* Bezy & Flores-Villela, 1999	LC (?)	H (16)	P	1	2
**Order Squamata**					
**Suborder Serpentes**					
**Boidae**					
*Boa sigma* (Smith, 1943)	NE	M (10)	NL	1	6
**Colubridae**					
*Conopsis nasus* (Günther, 1858)	LC (=)	M (11)	NL	1	5
*Drymarchon melanurus* (Duméril, Bibron & Duméril, 1854)	LC (=)	L (6)	NL	4	12
*Drymobius margaritiferus* (Schlegel, 1837)	LC (=)	L (6)	NL	4	10
*Gyalopion canum* Cope, 1861	LC (=)	L (9)	NL	2	4
*Gyalopion quadrangulare* (Günther, 1893)	LC (=)	M (11)	Pr	2	3
*Lampropeltis alterna* (Brown, 1901)	LC (=)	H (14)	A	2	3
*Lampropeltis californiae* (Blainville, 1835)	LC (=)	M (10)	NL	2	5
*Lampropeltis greeri* Webb, 1961	NE	NE	NL	0	EN
*Lampropeltis knoblochi* Taylor, 1940	LC (=)	H (14)	NL	2	1
*Lampropeltis mexicana* (Garman, 1884)	LC (=)	H (15)	A	1	5
*Lampropeltis polyzona* Cope, 1860	LC (?)	M (11)	NL	1	9
*Lampropeltis splendida* (Baird & Girard, 1853)	LC (=)	M (12)	NL	2	3
*Lampropeltis webbi* Bryson, Dixon & Lazcano, 2005	DD	H (16)	NL	0	EN
*Leptophis diplotropis* (Günther, 1872)	LC (=)	H (14)	A	1	8
*Masticophis bilineatus* Jan, 1863	LC (=)	M (11)	NL	2	6
*Masticophis flagellum* Shaw, 1802	LC (=)	L (8)	A	2	9
*Masticophis mentovarius* (Duméril, Bibron & Duméril, 1854)	LC (=)	L (6)	A	3	11
*Masticophis taeniatus* (Hallowell, 1852)	LC (=)	M (10)	NL	2	3
*Mastigodryas cliftoni* (Hardy, 1964)	DD (?)	H (14)	NL	1	4
*Opheodrys vernalis* (Harlan, 1827)	LC (=)	H (14)	NL	2	2
*Oxybelis microphthalmus* Barbour & Amaral, 1926	NE	NE	NL	2	9
*Pituophis catenifer* Blainville, 1835	LC (=)	L (9)	NL	2	8
*Pituophis deppei* (Duméril, 1853)	LC (=)	H (14)	A	1	7
*Pseudoficimia frontalis* (Cope, 1864)	LC (=)	M (13)	NL	1	7
*Rhinocheilus lecontei* Baird & Girard, 1853	LC (=)	L (8)	NL	2	8
*Salvadora bairdii* Jan & Sordelli, 1860	LC (=)	H (15)	Pr	1	8
*Salvadora deserticola* Schmidt, 1940	NE	H (14)	NL	2	5
*Salvadora grahamiae* Baird & Girard, 1853	LC (=)	M (10)	NL	2	6
*Salvadora mexicana* (Duméril, Bibron & Duméril, 1854)	LC (=)	H (15)	Pr	1	5
*Senticolis triaspis* (Cope, 1866)	LC (=)	L (6)	NL	4	11
*Sonora aemula* (Cope, 1879)	NT (=)	H (16)	Pr	1	2
*Sonora mutabilis* Stickel, 1943	LC (?)	H (14)	NL	1	5
*Sonora semiannulata* Baird & Girard, 1853	LC (=)	L (5)	NL	2	4
*Sympholis lippiens* Cope, 1862	DD (?)	H (14)	NL	1	3
*Tantilla bocourti* (Günther, 1895)	LC (?)	L (9)	NL	1	8
*Tantilla hobartsmithi* Taylor, 1936	LC (=)	M (11)	NL	2	4
*Tantilla wilcoxi* Stejneger, 1902	LC (=)	M (10)	NL	2	3
*Tantilla yaquia* Smith, 1942	LC (=)	M (10)	NL	2	4
*Trimorphodon lambda* Cope, 1886	LC (=)	M (13)	NL	2	4
*Trimorphodon paucimaculatus* Taylor, 1936	NE	H (15)	NL	1	5
*Trimorphodon tau* Cope, 1870	LC (=)	M (13)	NL	1	8
*Trimorphodon vilkinsonii* Cope, 1886	LC (=)	H (15)	A	2	3
**Dipsadidae**					
*Diadophis punctatus* (Linnaeus, 1766)	LC (=)	L (4)	NL	2	7
*Geophis dugesii* Bocourt, 1883	LC (?)	M (13)	NL	1	3
*Hypsiglena affinis* Boulenger, 1894	NE	H (14)	Pr	1	3
*Hypsiglena chlorophaea* Cope, 1860	LC (=)	L (8)	Pr	2	3
*Hypsiglena jani* Dugès, 1866	LC (=)	L (6)	Pr	2	6
*Hypsiglena torquata* (Günther, 1860)	LC (=)	L (8)	Pr	1	5
*Leptodeira maculata* (Hallowell, 1861)	LC (=)	L (7)	Pr	1	9
*Leptodeira punctata* (Peters, 1866)	LC (?)	H (17)	NL	1	4
*Leptodeira splendida* Günther, 1895	LC (?)	H (14)	NL	1	6
*Manolepis putnami* (Jan, 1863)	LC (=)	M (13)	NL	1	6
*Rhadinaea hesperia* Bailey, 1940	LC (=)	M (10)	Pr	1	7
*Rhadinaea laureata* (Günther, 1868)	LC (?)	M (12)	NL	1	3
*Rhadinaea taeniata* (Peters, 1863)	LC (=)	M (13)	NL	1	6
*Tropidodipsas repleta* Smith, Lemos-Espinal, Hartman & Chiszar, 2005	DD (?)	H (17)	NL	1	2
**Elapidae**					
*Micruroides euryxanthus* (Kennicott, 1860)	LC (=)	H (15)	A	2	3
*Micrurus distans* (Kennicott, 1860)	LC (=)	H (14)	Pr	1	7
*Micrurus proximans* Smith & Chrapliwy, 1958	LC (?)	H (18)	Pr	1	4
**Leptotyphlopidae**					
*Rena humilis* Baird &Girard, 1853	LC (=)	L (8)	NL	2	9
**Natricidae**					
*Storeria storerioides* (Cope, 1865)	LC (=)	M (11)	NL	1	6
*Thamnophis cyrtopsis* (Kennicott, 1860)	LC (=)	L (7)	A	4	10
*Thamnophis elegans* (Baird & Girard, 1853)	LC (=)	H (14)	A	2	2
*Thamnophis eques* (Reuss, 1834)	LC (=)	L (8)	A	2	7
*Thamnophis errans* Smith, 1942	LC (?)	H (16)	NL	0	EN
*Thamnophis foxi* Rossman & Blaney, 1968	DD (?)	H (16)	Pr	0	EN
*Thamnophis marcianus* (Baird & Girard, 1853)	LC (?)	M (10)	A	4	9
*Thamnophis melanogaster* (Peters, 1864)	EN (↓)	H (15)	A	1	5
*Thamnophis nigronuchalis* Thompson, 1957	DD (?)	M (12)	Pr	0	EN
*Thamnophis pulchrilatus* (Cope, 1885)	LC (?)	H (15)	NL	1	6
*Thamnophis scaliger* (Jan, 1863)	VU (↓)	H (15)	A	1	4
*Thamnophis sirtalis* (Linnaeus, 1758)	LC (=)	H (14)	Pr	2	2
*Thamnophis unilabialis* Tanner, 1985	NE	NE	NL	1	2
*Thamnophis validus* (Kennicott, 1860)	LC (=)	M (12)	NL	1	5
**Typhlopidae**					
*Indotyphlops braminus* (Daudin, 1803)					IN
**Viperidae**					
*Agkistrodon bilineatus* (Günther, 1863)	NT (↓)	M (11)	Pr	3	6
*Crotalus aquilus* Klauber, 1952	LC (↓)	H (16)	Pr	1	4
*Crotalus atrox* Baird & Girard, 1853	LC (=)	M (9)	Pr	2	9
*Crotalus basiliscus* (Cope, 1864)	LC (=)	H (16)	Pr	1	6
*Crotalus lepidus* (Kennicott, 1861)	LC (=)	M (12)	Pr	2	4
*Crotalus molossus* Baird & Girard, 1853	LC (=)	L (8)	Pr	2	8
*Crotalus polystictus* (Cope, 1865)	LC (↓)	H (16)	Pr	1	4
*Crotalus pricei* Van Denburgh, 1895	LC (?)	H (14)	Pr	2	2
*Crotalus scutulatus* (Kennicott, 1861)	LC (=)	M (11)	Pr	2	7
*Crotalus stejnegeri* Kennicott, 1859	VU (↓)	H (17)	A	1	2
*Crotalus tigris* Kennicott, 1859	LC (=)	H (16)	Pr	2	3
*Crotalus willardi* Meek, 1905	LC (=)	M (13)	Pr	2	1
**Order Testudines**					
**Emydidae**					
*Chrysemys picta* (Schneider, 1783)	LC (=)	H (14)	A	2	2
*Terrapene nelsoni* Stejneger, 1925	DD	H (18)	Pr	1	3
*Trachemys scripta* (Thunberg, 1792)					IN
**Geoemydidae**					
*Rhinoclemmys pulcherrima* (Gray, 1855)	NE	L (8)	A	3	5
**Kinosternidae**					
*Kinosternon hirtipes* (Wagler, 1830)	LC (↓)	M (10)	Pr	2	6
*Kinosternon integrum* LeConte, 1854	LC (=)	M (11)	Pr	1	9
*Kinosternon sonoriense* LeConte, 1854	NT (?)	H (14)	P	2	3

**Table 2:** Summary of the number of species shared between the Sierra Madre Occidental and neighboring biogeographic provinces (not including introduced species). The percent of the Sierra Madre Occidental shared by neighboring provinces are given in parentheses. Total refers to the number of species found in the Sierra Madre Occidental and three neighboring provinces (i.e., regional species pool) and the number in parentheses in this column is the percent of the regional species pool found in the Sierra Madre Occidental. - indicates either the Sierra Madre Occidental or their neighboring province has no species in the taxonomic group, or none of that specific taxon is shared between the provinces, thus no value for shared species is provided. Abbreviations are as follows: SMO (Sierra Madre Occidental); TVB (Transvolcanic Belt); CD (Chihuahuan Desert); and Pacific (Pacific Lowlands).

	SMO	TVB	CD	Pacific	Total
Class Amphibia	57	36 (63.2)	28 (49.1)	34 (59.6)	212 (26.9)
Order Anura	52	34 (65.4)	26 (50)	34 (65.4)	144 (36.1)
Bufonidae	13	6 (46.2)	8 (61.5)	8 (61.5)	22 (59.1)
Centrolenidae	-	-	-	-	1 (0)
Craugastoridae	6	4 (66.7)	2 (33.3)	4 (66.7)	17 (35.3)
Eleutherodactylidae	6	3 (50)	-	4 (66.7)	31 (19.4)
Hylidae	9	8 (88.9)	6 (66.7)	7 (77.8)	38 (23.7)
Leptodactylidae	1	1 (100)	1 (100)	1 (100)	3 (33.3)
Microhylidae	3	2 (66.7)	1 (33.3)	3 (100)	4 (75)
Ranidae	12	8 (66.7)	6 (50)	6 (50)	24 (50)
Rhinophrynidae	-	-	-	-	1 (0)
Scaphiopodidae	2	2 (100)	2 (100)	1 (50)	3 (66.7)
Order Caudata	5	2 (40)	2 (40)		66 (7.6)
Ambystomatidae	3	1 (33.3)	1 (33.3)	-	17
Plethodontidae	2	1 (50)	1 (50)	-	49
Order Gymnophiona					2
Dermophiidae	-	-	-	-	2
Class Reptilia	160	95 (58.8)	98 (61.3)	85 (53.1)	529 (30.2)
Order Crocodylia	-	-	-	-	2 (0)
Alligatoridae	-	-	-	-	1 (0)
Crocodylidae	-	-	-	-	1 (0)
Order Squamata	154	90 (58.4)	94 (61)	81 (52.6)	490 (31.4)
Suborder Lacertilia	67	32 (47.8)	39 (58.2)	32 (47.8)	232 (28.9)
Anguidae	5	3 (60)	4 (80)	2 (40)	14 (35.7)
Anolidae	1	1 (100)	1 (100)	1 (100)	20 (5)
Bipedidae	-	-	-	-	2 (0)
Corytophanidae	-	-	-	-	3 (0)
Crotaphytidae	2	-	-	-	4 (50)
Dibamidae	-	-	-	-	1 (0)
Diploglossidae	-	-	-	-	2 (0)
Eublepharidae	1	-	1 (100)	1 (100)	5 (20)
Gymnophthalmidae	-	-	-	-	1 (0)
Helodermatidae	3	1 (33.3)	-	3 (100)	4 (75)
Iguanidae	2	1 (50)	1 (50)	2 (100)	8 (25)
Phrynosomatidae	35	18 (51.4)	25 (71.4)	14 (40)	101 (34.7)
Phyllodactylidae	2	1 (50)	-	2 (100)	10 (20)
Scincidae	7	3 (42.9)	3 (42.9)	4 (57.1)	22 (31.8)
Sphaerodactylidae	-	-	-	-	3 (0)
Teiidae	8	3 (37.5)	4 (50)	3 (37.5)	21 (28.6)
Xantusidae	1	1 (100)	-	-	10 (10)
Xenosauridae	-	-	-	-	1 (0)
Suborder Serpentes	87	58 (66.7)	55 (63.2)	49 (56.3)	258 (33.7)
Boidae	1	1 (100)		1 (100)	3 (33.3)
Colubridae	42	25 (59.5)	29 (69)	26 (61.9)	93 (45.2)
Dipsadidae	14	12 (85.7)	9 (64.3)	9 (64.3)	70 (20)
Elapidae	3	3 (100)	1 (33.3)	3 (100)	15 (20)
Leptotyphlopidae	1	1 (100)	1 (100)	1 (100)	13 (7.7)
Loxocemidae	-	-	-	-	1 (0)
Natricidae	14	8 (57.1)	9 (64.3)	3 (21.4)	24 (58.3)
Typhlopidae	-	-	-	-	1 (0)
Viperidae	12	8 (66.7)	6 (50)	6 (50)	38 (31.6)
Order Testudines	6	4 (66.7)	4 (66.7)	4 (66.7)	37 (16.2)
Cheloniidae	-	-	-	-	4 (0)
Dermochelyidae	-	-	-	-	1 (0)
Emydidae	2	1 (50)	1 (50)	1 (50)	13 (15.4)
Geoemydidae	1	1 (100)	-	1 (100)	2 (50)
Kinosternidae	3	2 (66.7)	3 (100)	2 (66.7)	13 (23.1)
Testudinidae	-	-	-	-	3 (0)
Trionychidae	-	-	-	-	1 (0)
Total	217	131 (60.4)	126 (58.1)	119 (54.8)	741 (29.3)

**Table 3:** Summary of native species present in the Sierra Madre Occidental biogeographic province of Mexico by family, order or suborder, and class. Status summary indicates the number of species found in each IUCN [21] conservation status in the order DD, LC, VU, NT, EN, and CR [21] (see Table 1 for abbreviations; in some cases, species have not been assigned a status by the IUCN and therefore these may not add up to the total number of species in a taxon). Mean EVS ($\overset{-}{x}$) is the mean Environmental Vulnerability Score; scores ≥ 14 are considered high vulnerability [37,38] and category of risk in Mexico according to SEMARNAT [36] in the order NL, Pr, A, and P (see Table 1 for abbreviations).

Scientific Name	Genera	Species	IUCN	$\overset{-}{x}$ EVS	SEMARNAT
			DD, LC, NT, VU, EN, CR		NL, Pr, A, P
Class Amphibia					
Order Anura	17	52	1, 45, 2, 3, 1, 0	10.5	38, 12, 2, 0
Bufonidae	3	13	0, 13, 0, 0, 0, 0	10.3	12, 1, 0, 0
Craugastoridae	1	6	0, 6, 0, 0, 0, 0	12.5	5, 1, 0, 0
Eleutherodactylidae	1	6	0, 2, 1, 1, 1, 0	16.6	4, 2, 0, 0
Hylidae	5	8	0, 8, 0, 0, 0, 0	8.6	7, 1, 0, 0
Leptodactylidae	1	1	0, 1, 0, 0, 0, 0	6	1, 0, 0, 0
Microhylidae	2	3	0, 3, 0, 0, 0, 0	6.3	2, 1, 0, 0
Phyllomedusidae	1	1	0, 1, 0, 0, 0, 0	11	1, 0, 0, 0
Ranidae	1	12	1, 9, 1, 2, 0, 0	10.8	4, 6, 2, 0
Scaphiopodidae	2	2	0, 2, 0, 0, 0, 0	4.5	2, 0, 0, 0
Order Caudata	2	5	1, 3, 0, 1, 0, 0	12.5	2, 2, 1, 0
Ambystomatidae	1	3	1, 2, 0, 0, 0, 0	12.7	1, 2, 0, 0
Plethodontidae	1	2	0, 1, 0, 1, 0, 0	12	1, 0, 1, 0
Subtotal	19	57	2, 48, 2, 4, 1, 0	10.7	40, 14, 3, 0
Class Reptilia					
Order Squamata	43	154	14, 115, 3, 2, 1, 0	12.5	96, 36, 21, 1
Suborder Lacertilia	11	67	8, 46, 1, 0, 0, 0	13.1	50, 10, 6, 1
Anguidae	3	5	1, 3, 0, 0, 0, 0	11.8	2, 3, 0, 0
Anolidae	1	1	0, 1, 0, 0, 0, 0	13	1, 0, 0, 0
Crotaphytidae	1	2	0, 2, 0, 0, 0, 0	12.5	1, 0, 1, 0
Eublepharidae	1	1	0, 1, 0, 0, 0, 0	17	1, 0, 0, 0
Helodermatidae	1	3	0, 2, 1, 0, 0, 0	13	1, 0, 2, 0
Iguanidae	1	2	0, 2, 0, 0, 0, 0	17	1, 0, 1, 0
Phrynosomatidae	5	35	3, 24, 0, 0, 0, 0	13.1	31, 2, 2, 0
Phyllodactylidae	1	2	0, 1, 0, 0, 0, 0	15	2, 0, 0, 0
Scincidae	1	7	3, 3, 0, 0, 0, 0	13.1	4, 3, 0, 0
Teiidae	1	8	1, 6, 0, 0, 0, 0	13	6, 2, 0, 0
Xantusidae	1	1	0, 1, 0, 0, 0, 0	16	0, 0, 0, 1
Suborder Serpentes	32	87	6, 69, 2, 2, 1, 0	12	46, 26, 15, 0
Boidae	1	1	0, 0, 0, 0, 0, 0	10	1, 0, 0, 0
Colubridae	17	42	3, 34, 1, 0, 0, 0	11.5	31, 4, 7, 0
Dipsadidae	7	14	1, 12, 0, 0, 0, 0	11.1	8, 6, 0, 0
Elapidae	1	3	0, 3, 0, 0, 0, 0	15.7	0, 2, 1, 0
Leptotyphlopidae	1	1	0, 1, 0, 0, 0, 0	8	1, 0, 0, 0
Natricidae	3	14	2, 9, 0, 1, 1, 0	14	5, 3, 6, 0
Viperidae	2	12	0, 10, 1, 1, 0, 0	13.3	0, 11, 1, 0
Order Testudines	4	6	1, 3, 1, 0, 0, 0	12.5	0, 3, 2, 1
Emydidae	2	2	1, 1, 0, 0, 0, 0	16	0, 1, 1, 0
Geoemydidae	1	1	0, 0, 0, 0, 0, 0	8	0, 0, 1, 0
Kinosternidae	1	3	0, 2, 1, 0, 0, 0	11.7	0, 2, 0, 1
Subtotal	47	160	15, 118, 4, 2, 1, 0	12.5	95, 39, 23, 2
Total	66	217	17, 164, 6, 6, 2, 0	12	135, 53, 26, 2

## Data Availability

All of the data that support the findings of this study are available in the main text.
